# Comparative Transcriptomes and EVO-DEVO Studies Depending on Next Generation Sequencing

**DOI:** 10.1155/2015/896176

**Published:** 2015-10-12

**Authors:** Tiancheng Liu, Lin Yu, Lei Liu, Hong Li, Yixue Li

**Affiliations:** ^1^Key Laboratory of Systems Biology, Institute of Biochemistry and Cell Biology, Shanghai Institutes for Biological Sciences, Chinese Academy of Sciences, 320 Yueyang Road, Shanghai 200031, China; ^2^Key Laboratory of Contraceptive Drugs and Devices of National Population and Family Planning Commission of China, Shanghai Institute of Planned Parenthood Research, 2140 Xietu Road, Shanghai 200032, China; ^3^Shanghai Center for Bioinformation Technology, 1278 Keyuan Road, Shanghai 201203, China

## Abstract

High throughput technology has prompted the progressive omics studies, including genomics and transcriptomics. We have reviewed the improvement of comparative omic studies, which are attributed to the high throughput measurement of next generation sequencing technology. Comparative genomics have been successfully applied to evolution analysis while comparative transcriptomics are adopted in comparison of expression profile from two subjects by differential expression or differential coexpression, which enables their application in evolutionary developmental biology (EVO-DEVO) studies. EVO-DEVO studies focus on the evolutionary pressure affecting the morphogenesis of development and previous works have been conducted to illustrate the most conserved stages during embryonic development. Old measurements of these studies are based on the morphological similarity from macro view and new technology enables the micro detection of similarity in molecular mechanism. Evolutionary model of embryo development, which includes the “funnel-like” model and the “hourglass” model, has been evaluated by combination of these new comparative transcriptomic methods with prior comparative genomic information. Although the technology has promoted the EVO-DEVO studies into a new era, technological and material limitation still exist and further investigations require more subtle study design and procedure.

## 1. Introduction

Evolutionary developmental biology (EVO-DEVO) studies how the dynamics of development affects the phenotypic variation arising from genetic variation and its correlation with phenotypic evolution. In this subject there is a central issue, which is the most conserved period or the crucial section during the entire developmental process of an organism. To solve this issue, morphological studies, which are the major approach in developmental biology, have been conducted on different species in past years. However, these traditional observation methods are not sufficient for the requirement of precise quantification analysis. In such a demand, comparative transcriptomic studies have been utilized in these studies and generate some models about the evolutionary pressure of embryonic development.

Next generation sequencing technology has largely improved the scale of comparative genomics studies by the high throughput detection of gene sequences, which makes the assembly of new genome easy. Besides, not only have the comparative genomics studies with case-control studies design reached a new level, but also the evolution studies based on genome sequences of multiple species have been feasible. When comparative transcriptomic studies of embryo development are equipped with this powerful tool, it also has generated unprecedented revolution in EVO-DEVO field and improved the resolution from macro to micro view. Several strategies have been proposed to illustrate the existing models of selective pressure acting on embryonic development, which provide further understanding for the divergence of morphogenesis.

## 2. Comparative Genomic and Comparative Transcriptomic Study

### 2.1. Comparative Genomics: From Case-Control to Multiple Species

Case-control study design is widely adopted in epidemiology for investigating the relationship between disease and exposure and it is the initial principle of comparative studies. In genomic studies, this design works efficiently for the comparison of two objects and it aims to illustrate relationship between the phenotypic difference and the genetic difference. Phenotypic difference stands for disease while genetic difference stands for exposure in terms of epidemiology. From the genomic opinion, the genetic differences were variants between case and control samples. Many genome-wide association analysis studies (GWAS) also employ the case-control study design to examine the potential effects of genetic variants among populations [1–3], which has promoted the understanding of many kinds of diseases [4].

**Figure 1:**
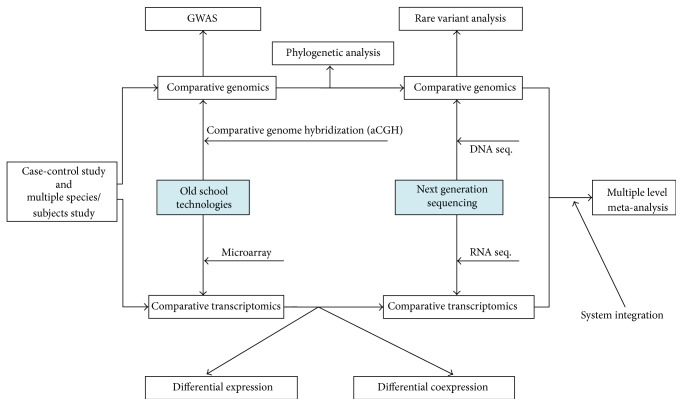
Illustration of the comparative genomics and comparative transcriptomics based on case/control study design are conducted with old school technologies and next generation sequencing technology. This figure shows the main concepts in the first part of this paper.

During the microarray era, there are many comparative genomic studies which adopted array comparative genome hybridization (aCGH) technology to determine copy number variations (CNVs) [5] or oligonucleotide array technology to investigate single nucleotide polymorphisms (SNPs) [6]. Along with the birth of next generation sequencing (NGS) technology, these microarray based technologies have been replaced as they are not convenient to acquire any interested genome sequences of organism as sequencing. Besides some de novo sequencing works [7, 8], most studies tend to conduct the resequencing procedure with case-control study design [9, 10]. It is meaningful to sequence comparable subjects and detect the underlying genetic difference, such as the fact that Atanur et al. have discovered the likely cellular basis of hypertension by comparing the genome of SHR strain rat with BN rat reference genomes [11]. The Bactrian Camels Genome Sequencing and Analysis Consortium have identified characters of domestication in camel by comparing the heterozygosity rate of wild and domestic Bactrian camels [12]. In the light of NGS technology, GWAS also have transformed from common variants to rare variants (Figure 1).

The case-control study design is narrow sense of comparative genomic as it is unnecessary to limit the comparison between two objects. Multiple objects comparison involves intraspecies comparison and interspecies comparison designed for different purpose. Intraspecies comparison intends to discover the strains diversity for specific species or the variation in population for certain species. The STAR Consortium has used SNP array to illustrate the diverse genetic background of different inbred laboratory rat strains [13], and the follow-on work has been conducted by Atanur et al. depending on next generation sequencing [14]. Similar study has been conducted to study the artificial selection during chicken domestication [15]. Navin et al. have applied single-nucleus sequencing to investigate tumor population structure and evolution in human breast cancer [16]. Actually the comparison between multiple objects is crucial especially in evolutionary analysis [17]. The interspecies comparison focuses on evolutionary analysis which examines the selective constraints acting on sequence of genome. Zhang et al. have compared the expansion or contraction of gene families between two bats and other eight mammalian species to reveal the genetic and evolutionary background for the functional characters of bat [18]. Besides, many studies trace certain species in phylogeny based on similarity of ortholog sequences between the studied and several known species [12, 19–21].

### 2.2. Comparative Transcriptomic Methods: Differential Expression and Differential Coexpression

Sequencing technology gives great impetus to comparative genomic studies, while the sequencing object is far beyond the DNA sequence. Capturing transcripts in cell makes the RNAs also available to the sequencing platform, which is used for quantification of the expression or detection of alternative splicing events. Sequencing technology also has improved comparative transcriptomic studies as it has produced plenty RNA data for transcriptomic investigations (Figure 1).

The traditional comparative transcriptomics are also based on the case-control study design, in which the gene expressions of several samples for each group are measured and statistical tests are adopted to examine the differential gene expression between case and control subjects. The differentially expressed genes are considered to be associated with phenotypic divergence between compared objects and they have potential to be the candidate biomarker of case situation. Recently, in the light of the high throughput technology such as microarray or RNA sequencing, expressions of 10 thousand genes can be detected at the same time. The big advance expands the scale of expression detection but also leads to the problem of multiple comparisons. The problem reduces statistical power so that several genes with expression change are neglected. Beside the problem of multiple comparisons, differential expression analysis also is defectiveness in following network analysis. For instance, in order to study their functions, the differentially expressed genes are always aligned onto the interaction network which is built by prior knowledge of protein interactions, which will not discover the new connections of genes.

Considering these deficiencies, it is necessary to further mine the information hiding in the expression matrix, which prompts the birth of differential coexpression analysis focusing on the switch of the links between genes rather than the changes of expression values for a single gene between samples [22]. In the system of organism, genes are organized into networks rather than separated, and genes are always linked to regulators such as TF, which lead to two genes regulated by the same TF exhibiting correlation in their expression profiles. The correlation of gene pair varies in different condition because the regulation relationship between genes will switch when the organism is exposed to different situation. Based on this principle, with several samples measured in case and control group, respectively, we are able to measure the correlation coefficients of every gene pair in each group. By comparing these correlation coefficients between case and control groups, the differential coexpression gene pairs can be identified. The differential coexpression approach not only complements the result of differential expression analysis but also enables the identification of rewiring events in the gene regulation network (GRN).

### 2.3. Annotation of Regulatory Element: The Integration of Genomics and Transcriptomics

Next generation sequencing not only prompts the efficiency of genomic research [23] but also facilitates the construction of genomic libraries for populations [24]. However, for the accumulation of sequences we have found abstruse information associated with biological function underlining the genome sequences. In order to further understand the biological function, we need to analyze the regulatory mechanism of the genomic elements, which lead to the transformation from comparative genomics to comparative transcriptomics. In such kind of demand, the Encyclopedia of DNA Elements (ENCODE) project and Model Organism Encyclopedia of DNA Elements (modENCODE) project have born and focus on annotation of the regulatory elements in genomes including human, mouse, fly, and worm [25–28]. They have profiled several crucial features in transcriptome such as the binding sites of transcription factors (TFs), epigenomic modifications, and gene expression levels for these species, which provide plentiful datasets for transcriptomic analysis. Depending on the profiles of epigenomic modifications, Ernst et al. have classified the human chromatin into 15 kind states which represent the activated conditions [29]. Based on the binding of TFs, Yip et al. have used machine learning approach to discriminate genomic regions [30]. By correlating epigenomic modifications on the cis-regulation region and gene expression in each species, Cheng et al. have proved that gene expression is predictable by chromatin features in fly and worm [31]; at the same time Dong et al. also model gene expression levels by histone modification profiles in human cell lines [32]. Finally, a universal model has been proved for epigenomic modifications on cis-regulation region to predict gene expression in these three species [33].

Although not every organization is able to produce such diversiform datasets, the integration of genomic information with transcriptomic information has been adopted by many investigators. These studies have made difference in understanding the regulated elements in genomic sequences. The integration of multiple levels, which also represents the trend of omic study nowadays, is based on the hypothesis that switches in higher level will influence the lower level which is coordinated with the Central Dogma. In other words, it proposes that the genomic mutations in gene sequence will lead to the change of expression level of downstream genes. Applying this principle, Akavia et al. have developed an algorithm to identify the casual genetic aberrations in cancer through associating chromosomal copy number variation (CNV) and gene expression data [34]. Kim et al. have identified potential causal genes by combining the expression Quantitative Trait Loci (eQTL) analysis with pathway information [35]. The integration of multiple level data not only increases the utilization of datasets but also ensures the reliability of result. It is wildly adopted in biological investigation nowadays, especially in studies of cancer which are conducted by The Cancer Genome Atlas (TCGA) [36, 37].

In summary, as a branch of computational science, bioinformatics has been promoted by the coming of big data era. More and more datasets will be generated by consortium like ENCODE and TCGA, and the meta-analysis will still be the trend in future.

## 3. EVO-DEVO Studies for Understanding the Morphological Diversity of Species

### 3.1. From Macro to Micro: Morphological Study to Gene Study

The development process of animals has been proposed to be under stringent selective pressure in order to ensure the precision of the process. The evolutionary pressure constrains the phenotypic diversity of embryo for different organism at certain degree, which leads to the morphological similarities at some stages of embryo development for different species. And the extents of embryo similarities between species are diverse during development process, which enables development biologists to examine the fluctuations of evolutionary pressure acting on different embryo stages. Development biologists have used this embryo morphological comparison method to study organism development for many years. For instance, von Baer's third law has proposed that the earlier development stages are highly similar between different species and the embryos gradually present divergence from each other during ontogeny [38]. Ontogenic stages stand for developmental process in contrast with phylotypic period, in which the morphology of embryos from different species represents such a high similarity that these development stages are considered to recapture the phylogeny during evolution. As above mentioned, discriminating ontogenic stages from phylotypic stages are central issue in the EVO-DEVO studies. However, a defect of the morphological comparison method is that it is difficult to quantize the morphological features, which would cause problem using nonquantitative morphological characteristics to evaluate the quantitative degree of conservation. And the stages with certain morphological characters are various in multiple phylum, which limits the morphological comparison which only can be conducted in a certain phylum. Taken together, these will confuse the definite detection of selective constraints acting on stages in multiple species.

Along with the advance of the technologies in molecular biology, development biologists have been able to analyze the development stages from microcosmic view. For instance, Duboule has found that the expression of Hox genes is a feature of the phylotypic stages [39]. The information from molecular comparison provides more precise identification of patterns for ontogenic stages and phylotypic stages in embryo development as it can produce the quantitative information. Until recently, new high throughput technologies, which possess more accurate quantitative characteristics, have been applied to development studies. Depending on microarray, Vassena et al. have examined gene expression in human preimplantation development [40], and the expression profile of whole development time series for zebra fish has been inspected by Domazet-Lošo and Tautz [41]. RNA sequencing method also has been adopted to address the expression profile of development in multiple species including fly [28], worm [27], human, and mouse [42].

Advance in the technology enables the EVO-DEVO studies from macro to micro. From microcosmic view, development biologists would further decipher possible evolutionary mechanism underlying the hypothesis, which is more challenging and meaningful. These molecular level studies are thought to be superior compared with the morphological approaches, as the information of gene sequences is more close to the inherited entities compared with the morphology. However, it is still a controversial problem for the discrimination of ontogenic stages and phylotypic stages in embryo developmental process for multiple species. New high throughput technology has potential to distinguish these stages depending on comparative transcriptomic analysis, which would further contribute to understanding the underlying molecular and evolutionary mechanism of development.

### 3.2. Two Controversial Models about the Constraints on Development

Above we have mentioned the controversial partition about the ontogenic stages and phylotypic stages during development stages, which can be illustrated as problem of defining phylotypic stages in certain period of development. Phylotypic stages are supposed to be development stages with high similarity among different species, in which the features of nature selection such as gene expression or gene sequence should present to be conserved. In ontogenic stages, species specific differentiation happens and features in these stages should be less conserved. In particular, because of their conserved feature, evolutionists, who intend to label these stages in certain developmental period for understanding the evolution of development, which is the central issue of EVO-DEVO studies, are also interested in phylotypic stages.

Organism developmental process can be classified into three dominant periods: earlier stages marked by the prominent event, zygote genome activation (ZGA) [42], middle stages when Hox genes start expression [43], and late stages in which morphological formation starts [44]. The late stages are unanimous to be the most nonconserved because embryos of different species already present diversity in these stages, whether morphological divergence or variations of gene expression. Although Tian et al. have found that the late stages show the strongest conservation and weakest evolvability in the slime mold* Dictyostelium* [45], these stages are still considered to be less conserved among most organisms, especially vertebrate. Besides this rare case of slime mold, two canonical evolutionary models of development have been proposed: the “funnel-like” model, in which it is supposed that the earlier embryo stages are the most conserved, and the “hourglass” model, in which the middle stages of development are imposed with the strongest evolutionary constraints [46].

**Figure 2:**
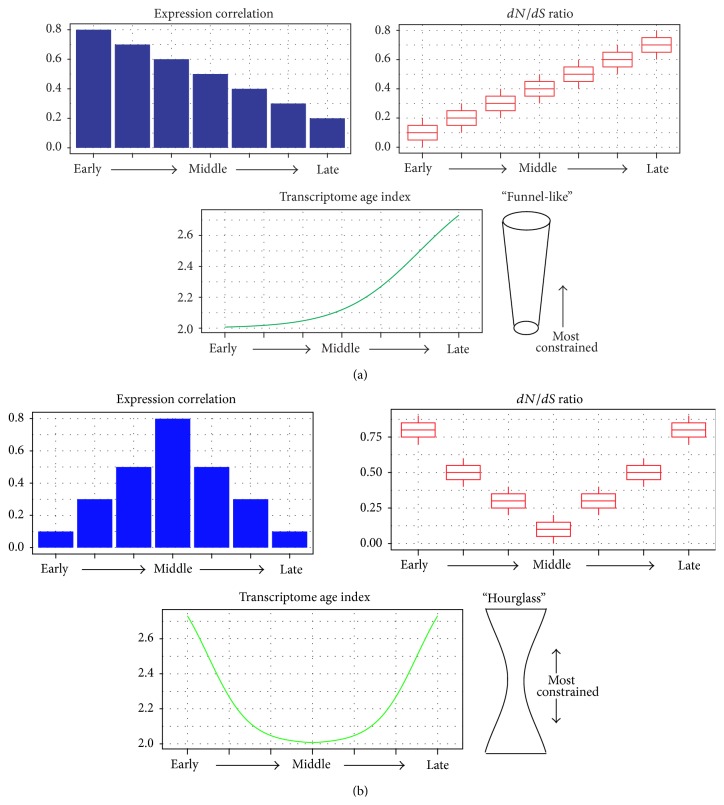
Illustration of the two major models about the selective pressure of embryonic development and their measurement. Pictorial charts in the right side stand for the “funnel-like” model and “hourglass” model. Histograms, boxplots, and lines from left to right stand for the three kinds of measurement of selective pressure based on transcriptomic data. In the figure of expression correlation, higher expression correlation means transcriptome similarity, which is the signature of conservation. In the figure of* dN*/*dS* ratio, lower* dN*/*dS* ratio means the conserved gene sequences. For the computation of transcriptome age index, ancient genes are labeled with small numbers while young genes are labeled with big numbers. Therefore, lower transcriptome age index of a stage means more ancient genes expressing in corresponding stages in the figure of transcriptome age index.

The “funnel-like” model, which describes the shape of selective constraints acting on development as a funnel (Figure 2), has been rooted in von Baer's third law. This law suggests that the selective constraints gradually decrease during the development and the earlier stages of development are under most stringent selective pressure. The development process starts from a single zygote cell, along with cell division occurring; it forms blastocyst which is composed of multiple cells with different fates. This process looks very similar to the evolution of creature, which starts from single cell to multiple cell. Therefore, phylotypic stages are thought to recapture the phylogeny in evolutional history and the development process is supposed to be an expand procedure from simple to complex. The earlier stages, which are considered to be simplex, should be exposed under strict selection so that the later developmental program can be subtly executed. This is in concordance with the developmental burden hypothesis [47], which has assumed that earlier elements in embryo are responsible for downstream development infrastructure so that earlier stages tend to be evolutionarily conserved.

The “hourglass” model, which assumes the mid-embryonic stages, shows the most stringent constraints and the shape of constraints looks like an hourglass with two wide sides and a narrow middle (Figure 2). This model initially depends on the functional importance and complicated regulation network of middle stages, in which the Hox genes express and the embryo forms body plans [39]. This fundamental process is considered to be such a crucial infrastructure in embryo development that perturbation during these stages will cause tremendous influence on organogenesis. In old school embryo morphological time, although some alternative models have been proposed [48–51], the hourglass model has been validated by observations of morphological traits in multiple vertebrate embryos [52–55]. In recent years, the embryo development stages have been profiled by parallel sequencing so that the hourglass can be examined in gene level. By comparing the expression profile in embryo development for turtle and chicken, Wang et al. have validated the hourglass model in development of these two species [21].

There are two major approaches in comparative transcriptomic studies to illustrate the hourglass model or funnel-like model, which we will discuss in later part (Figure 2).

## 4. Comparative Transcriptomics for the Embryo Developmental Studies

### 4.1. Correlation of Gene Expression Methods

Based on the case-control study design, an intuitive measure for the conservation between two objects is to compare the similarity of their gene expression. The comparison of expression profile is conducted on one-to-one ortholog genes, which maintain single copy and usually are considered to possess the same biological functions in corresponding species. Therefore, the expression pattern of one-to-one ortholog gene pair should present certain degree of similarity. In definition of this method, conservation is measured by computing the correlation coefficients between all pairs of expressed one-to-one ortholog genes in each developmental stage. The levels of conservation are determined depending on the summary of all the correlation coefficients in each stage. The high correlation coefficients of a certain stage indicate conserved gene expression in this stage, which should be considered under strong selective pressure (Figure 2). As comparative genomics studies can be conducted between or within species, the comparative transcriptomics studies also can illustrate the diversity of gene expression within species or between species. Kalinka et al. have used these comparative transcriptomics studies to examine the correlation of gene expression within six sequenced* Drosophila* species [56], and Ninova et al. have detected the correlation of microRNA expression within two divergent fruit flies [57]. Both of these studies prove the broad existence of hourglass model for multiple kinds of transcripts within* Drosophila* species. As evidence for the hourglass model holding between species, the study of Wang et al. has been conducted on two different species [21]. The study of Irie and Kuratani proves the common existence of hourglass in vertebrate by comparing the expression profiles of four species [46]. These pioneering investigations have successfully applied expression correlation approach in EVO-DEVO studies, which proves comparative transcriptomic approach is powerful in evolutionary study.

The transcriptomic similarity method also has some defects, such as the fact that the correlations are examined only depending on a part of the whole transcriptome (one-to-one ortholog genes) and the computation must be conducted on two subjects/species. One-to-one ortholog genes only account for part of expression signature in each one of the compared objects. Particularly for studies conducted between distant species, the proportion of one-to-one ortholog genes becomes even smaller. It results in loss of expression information, which will further affect the conclusion. In particular, the evolutionary distance between two objective organisms is not in direct proportion to the loss of expression information, which will cause the difficulties in different studies using pairs of species with various evolutionary distances. Besides the problem of losing information, another difficulty is the choice of corresponding development time points in paired organisms. Only development stages of two species are aligned in corresponding development time points; the correlation coefficients can be computed in each of the aligned stages. However, the developmental time varies between species, which makes it difficult to find the precise alignments of stages. To solve this problem, investigators have adopted enumeration method which computes the correlation coefficients between paired stages in all-to-all manner [7, 56]. Enumeration method handles the problem of corresponding stage choice, but it will introduce artificial decision especially in the case in which one species has multiple corresponding stages in the other species, such as the dual alignment in fly and worm development stages found by Gerstein et al. [33].

### 4.2. Evolutionary Indices Based Methods

We have discussed the comparative transcriptomic method based on the correlation and its two major limitations above. This approach presents an oversimplified procedure. It not only neglects the information of nonortholog genes but also does not utilize the prior knowledge. Prior knowledge of conservation is contained in the sequence of expressed genes during each developmental stage. Such kind of knowledge has been evaluated by prior comparative genomic studies [58]. For instance, each gene has unique date of birth in the phylogeny which means a specific gene has been born in certain ancient time. Age information of gene should be applied to studies. In the evolutionary indices based approach, first the gene expression of a certain species during embryonic development has been profiled. Then a specifically activated set of genes have been identified for each developmental stage and the age indices of each gene set are used to measure the conservation of corresponding stage. Depending on the age index of genes, Domazet-Lošo et al. have developed a phylostratigraphy approach to specify different phylostratum for genes expressing in ectoderm, endoderm, or mesoderm of* D. melanogaster* embryo [59]. The principle of phylostratigraphy approach is labeling ancient genes with small numbers and young genes with big numbers so that phylogenetic ages of genes are quantified. It also has been used to study the relationship between multicellularity and the origin of cancer [60]. Based on this approach, Domazet-Lošo et al. have further proposed a transcriptome age index (TAI), which combines phylostratigraphy and stage-specific gene expression information by multiplication, to evaluate the selective pressure on stages of zebra fish development [41]. Not only has this study proved the hourglass in zebra fish, but also another study of* Arabidopsis thaliana* embryogenesis, in which the conservation has also been measured by TAI, has showed the existence of hourglass in Plantage [61]. Depending on the transcriptomic information, TAI measures the relative proportion of ancient genes and young genes in a specific developmental stage (Figure 2). Such kind of approach represents the combination of prior comparative genomic knowledge with the gene expression information between different development stages within a species.

Compared with transcriptome similarity method, the evolutionary index based method has some significant advantages, such as the fact that it only requires expression profile of one species and makes full usage of prior knowledge. In particular, except for the gene age index, more evolutionary information can be retrieved from prior knowledge. For instance, the adaptive selections of genes can be traced by the nonsynonymous to synonymous substitution ratio (*dN*/*dS*) of sequences in specific phylogenetic clade, and genes with low* dN*/*dS* ratio are thought to be under selective pressure in certain species [62]. Besides, there are many expansions or contractions of gene families during the formation of each species, which lead to the copy number variations of homolog genes in different species [63]. Therefore, the states of gene duplication also imply diverse selective pressure on different genes for certain kind of species. Combining these two indices with the gene age index, Piasecka et al. have measured the transcriptomic conservation of embryonic development and evaluated the conservation of transcription regulation in zebra fish [64]. They completely reexamined conservation of development stages based on the expression profile of zebra fish embryogenesis, which is the same datasets adopted by Domazet-Lošo and Tautz [41]. Their result has showed the coexisting patterns of funnel model and hourglass model, as these evolutionary indices address different aspects of selective pressure and they are unable to make unanimous decision for either model. In addition, new method has been developed and tries to combine the evolutionary index with the gene expression for identification of conserved coexpression modules between species [65]. This method has been applied on the study carried out by Gerstein et al. [33], which has investigated the conservation of coexpression modules in development stages for worm and fly.

## 5. Discussion

As two major existing approaches of transcriptomic studies for EVO-DEVO, both of the correlation of gene expression method and the evolutionary indices based method show some advantages and defects (Table 1). Correlation of gene expression method can measure conservation inter-/intra-species/subjects while evolutionary indices based method combines age indices and evaluates conservation in a single subject. As the study of Piasecka et al. shows, these two approaches address different aspects of evolution so that combination of them would make a more comprehensive conclusion about the evolutionary model of embryonic development. We have summarized that the 3 major indices should be adopted to evaluate the model of development, for both hourglass and funnel model (Figure 2). These 3 measurements include gene expression correlation,* dN*/*dS* ratios, and transcriptome age index, which show different aspects of evolutionary selection. For instance, gene expression correlation stands for the similarity of paired transcriptome,* dN*/*dS* ratios show the selective pressure on gene sequences, and transcriptome age index combines the gene expression with phylogenetic age. These 3 measurements present significantly different patterns for each model. For instance, in hourglass model, the middle stages present the highest gene expression correlation and genes of these stages not only have conserved sequences but also are born in ancient time. In funnel-like model, these signatures present in the early stages of embryonic development (Figure 2).

Organism development is a cell expansion process which starts from single cell to multiple cells with different destinies. This procedure transforms from simple to complex in the view of the diversity of cell composition, which is in more concordance with von Baer's third law. However, nowadays more and more comparative transcriptomic researches support the hourglass model which proposes that the most conserved stages are in the middle period rather than the earlier period. The hourglass model is still not concluded as these comparative transcriptomic studies have technological limitation. For instance, except for the zygote, the rest of stages of embryo are composed of multiple cells, and the diversity of these embryonic cells increases along with the developmental time line. The RNA source for the comparative transcriptomic studies is extracted from embryo sample in multiple developmental time points and the RNA extractive is mixture of multiple cells. And along with development process, the RNA extractive includes more and more diverse RNAs from various cells. The different extent of RNA mixture at different development time points will affect the evolutionary conservation analysis results, as these comparative transcriptomic studies assume that every representative of different development stages is considered to be single and equivalent. Based on the single cell RNA sequencing dataset of human preimplantation embryo [66], we have showed even in the early stages that there are up- and downfluctuation of selective pressure [67]. However, these single cell RNA sequencing datasets only cover the early stages of embryo for some species [42, 66, 68].

Besides the technological limitation, there are inherent problems in the experimental materials. For instance, many studies, which try to illustrate that the hourglass model universally exists in multiple species, have been conducted on the model organism such as mouse, worm, and fly. Compared with normal organisms, these model organisms share some common features such as short generation period and quick developmental time, which represent a specific mechanism of development and will potentially bias the result model of evolutionary studies [54]. Along with the decreasing of sequencing cost, more organisms, especially those that have long development procedure, should be profiled with multiple object sequencing. Depending on the single cell RNA sequencing technology, the whole embryonic development of more species would be profiled. Moreover, precise studies should be designed to illustrate this problem and construct sophisticated models about the evolution of development.

## Figures and Tables

**Table 1:** Comparison of two detection approaches from different aspects.

	Sample	Prior knowledge	Advantages	Defects
Correlation of gene expression methods	Paired	No	Interspecies evaluation	Loss of information
Evolutionary indices based methods	One	Yes	Integration analysis	Single species evaluation
